# Portugal as a Holiday and Health Resort

**Published:** 1906-05-19

**Authors:** 


### PORTUGAL AS A HOLIDAY AND HEALTH RESORT.

The English, if not a wandering race, are certainly a travelling one; and there are some of us who have visited nearly every country in Europe, and yet to whom Portugal is *terra incognita*. There are several reasons for this. First of all, the land route is long and tiresome, and, we may add, expensive. From London to Paris, and from Paris to Hendaye, the train service is fairly good; but from the latter it is tedious, and the time of direct travelling is not less than sixty hours from London to Lisbon, the first-class ordinary single fare being about £11, and the Sud Express much more. To those who can face a sea voyage there are several routes open, such as the Royal Mail Line from Southampton and the Hall's Line from London. The latter is the cheapest, but the boats would be by most people considered small nowadays. Of late the Booth Line have offered many facilities for visiting the Peninsula. Their new steamers, the *Ambrose* and the *Anselm*, are large and well equipped in every way; and a pair of new six-thousand tonners will be on the station towards the end of the year. The steamers of this line leave Liverpool every ten days, and on the larger steamers, at any rate, there is always plenty of room between Liverpool and Lisbon. Unfortunately, they do not touch at any South of England port; and we would suggest that a trial be made of Dartmouth, as we believe that would have a decided influence in increasing the number of passengers. It is, however, possible to take the Southampton-Havre steamer and join the Booth Liner at the latter port. From Havre the steamer goes direct to Oporto, and passengers may leave here and take the train to Lisbon, or preferably to Pamphilosa; then to Luza, and from that station drive through the woods to Busacco, where is the Grand Hotel, a new building tacked on to an old monastery. It is in the midst of magnificent woods, in which there are many drives and sheltered walks; but it is high, about twelve hundred feet above the sea level, and in winter some people might think it too cold, especially as the hotel is insufficiently warmed for our English ideas of comfort. Or the traveller may spend a day or two in Oporto, the site of which is decidedly striking, and go on to Lisbon with the vessel. He will enjoy sailing along the Portuguese coast, and when the anchor is dropped in the Tagus he will be charmed with the appearance of the city which lies spread before him on the right bank of the river. It may remind him a little of Constantinople. The resemblance is there, faint and washed out perhaps, but still it is present, and the seven hills are more distinctly marked out than in the Turkish capital.

In Lisbon itself there are not many "sights" in the ordinary acceptation of the term. Of course, there are churches, and orphanages, and museums, and the visitor to the fruit and fish markets will find a seething mass of humanity well worth studying if his visit be made early.

From Lisbon a rather pretty train journey of fifteen miles along the coast will take him to Mont' Estoril. This little place, with S. Joad do Estoril and Estoril, form the Portuguese Riviera. Perhaps Cascaes ought to be included. Our visit was made in the month of March—that month so terribly hard for the invalid to bear in England—and here the roses were hanging in luxuriant garlands over the houses; the Judas trees were in full blossom, and the sun strong enough to make the shade from the palm trees welcome. Both the hotels at which English people are likely to stay at Mont' Estoril. Here, we think, the proprietors made a mistake. One at least ought to have been at Cascaes, which is in most respects infinitely prettier than Mont' Estoril, which has an unfinished, almost a neglected, look; and it will be some time before it can rival the Italian or French Riviera. Furthermore, as it lies along the west coast of Europe, it must necessarily get some share of Atlantic storms; but then it is three hundred miles further south than the French Riviera, so that it ought to have a milder climate, and what we have said about the vegetation proves that the average temperature must be high during the early spring months. For those who wish quietude, and who dislike fashionable resorts, Mont' Estoril has much to recommend it, and it may be incidentally remarked that both hotels are quite good, very clean, and the charges moderate.

Those who wish to go on to Cintra have their choice of two courses. They can return to Lisbon and take the train out, the distance being about eighteen miles. It is preferable to drive, and it may be done in an hour and a half, or less. The first part is nearly all uphill, and when the summit is reached Cintra is seen sleeping on the brow of the hill. Byron calls it the most delightful spot in Europe. That is a poet's imagination or exaggeration; but lovely it certainly is. At its back rise rugged hills, one peak crowned with the ruins of an old Moorish castle, and on another sits the Palace of the present King of Portugal. In the immediate neighbourhood of Cintra are cork woods, shrubberies, orange and lemon groves, shady walks, good roads, and an electric tram runs to the sea-coast about three miles off.

As to the class of invalids for whom Portugal is suitable, or as to the best time of the year to go, it is somewhat difficult to advise. Some of the hotels refuse to admit consumptives, so that, except in the very earliest stages, no one with that disease should try it, unless private rooms can be taken. Speaking generally, it ought to suit a large number of people who are not seriously ill; but who are scarcely able to bear our English winter with impunity. To this class it may be recommended, especially if they can do without the services of an English medical man, for these practitioners are rare in Portugal. During the early winter Mont' Estoril would be better than Cintra, as the latter is over six hundred feet above the sea-level; and Cintra, again, would be better than Busacco, which is twelve hundred feet; but in one or other of these the invalid ought to find a climate which suits him. It is said that Busacco has an average winter temperature of 50°, a spring temperature of 60°, and a summer one of 70°; but we fear these come out rather too formally to be quite reliable, and a little practical experience is the best guide. Besides, mere temperature is not everything. Our sensations of heat and cold depend so much upon whether the air around us is still or in motion. Our advice, then, to the traveller would be to take a Booth Line ticket for Tour F. The price of this is £18 10s. from London to London, and it includes hotel expenses for the trip, which, between sea and land, occupies a total of about twenty-six days. By these means he would visit Coimbra, Busacco, Cintra, Lisbon, etc.; but not Mont' Estoril which would have to be done from Lisbon; and he would have a general idea of Portugal, and could decide for himself which part would suit him best. This course would also free him from all difficulties as regards landing, conveyance of luggage, etc., as Messrs. Booth's representatives do all these for him, and, if our experience can be relied on, these services are very pleasantly rendered. A word might be said regarding Portuguese trains, postal arrangements, and cabs. It would almost seem as if the train service to Busacco had been designed to keep people away from the town. Four thousand reis for an hour and a half's drive is a sum the half of which would have satisfied an English cabman. People do not like waiting until the fifth day for their London letters when they might quite easily arrive on the third day. Sometimes English newspapers come in batches: at other times those of a certain date will arrive a day earlier than the papers of the preceding day, and so on. If the Portuguese authorities wish to induce any considerable number of English visitors these drawbacks should be remedied.

